# The Risk of Radiation Exposure to the Eyes of the Interventional Pain Physician

**DOI:** 10.1155/2011/609537

**Published:** 2011-05-17

**Authors:** David E. Fish, Andrew Kim, Christopher Ornelas, Sungchan Song, Sanjog Pangarkar

**Affiliations:** ^1^UCLA Medical Center, 1245 16th Street, Suite 220, Santa Monica, CA 90404, USA; ^2^Department of Physical Medicine and Rehabilitation, Pain Medicine Division, West Los Angeles Veteran's Administration Medical Center, UCLA, 11301 Wilshire Boulevard, Los Angeles, CA 90025, USA

## Abstract

It is widely accepted that the use of medical imaging continues to grow across the globe as does the concern for radiation safety. The danger of lens opacities and cataract formation related to radiation exposure is well documented in the medical literature. However, there continues to be controversy regarding actual dose thresholds of radiation exposure and whether these thresholds are still relevant to cataract formation. Eye safety and the risk involved for the interventional pain physician is not entirely clear. Given the available literature on measured radiation exposure to the interventionist, and the controversy regarding dose thresholds, it is our current recommendation that the interventional pain physician use shielded eyewear. As the breadth of interventional procedures continues to grow, so does the radiation risk to the interventional pain physician. In this paper, we attempt to outline the risk of cataract formation in the scope of practice of an interventional pain physician and describe techniques that may help reduce them.

## 1. Introduction

The use of medical radiation for diagnostic and therapeutic purposes continues to increase worldwide. Outside of inpatient radiology services, the use of fluoroscopy has increased and now encompasses multiple specialties. For the pain interventionist, there has been exponential growth of the number of fluoroscopy-guided procedures in the past decade [1]. With procedures such as epidural steroid injections, studies have demonstrated increased accuracy, precision, and patient safety with fluoroscopic guidance [2, 3]. Furthermore, the improvement of technology and expansion of more percutaneous and less open procedures appears to be increasing the use of fluoroscopy guidance for the pain interventionist. The increased use of these diagnostic tools is also paralleled in general radiology with CT scans and nuclear imaging, approaching 67 million and 18 million, respectively [4, 5]. The purpose of this article is to more closely evaluate the risk to the pain interventionist regarding lens opacification and cataract formation in the format of a narrative review. It is unclear how many interventionists engage in proper eye protection and precautions in the United States. However, with further analysis of the literature, it is the authors' intent to further elucidate these known risks. 

With the growing use of fluoroscopy for diagnosis and procedure-guidance, there has also been growing concern regarding occupational radiation exposure. The International Commission on Radiological Protection (ICRP) advises compliance of 3 main principles: justification, optimization, and dose limitation to help reduce unnecessary occupational radiation exposure [5]. ALARA (as low as reasonably achievable) is also a concept applied by healthcare professionals in regards to radiation safety. With the application of these concepts and other preventive measures, the practitioner can further minimize adverse effects of radiation exposure to oneself and others.

Multiple epidemiological studies from Hiroshima, Nagasaki, and children survivors from Chernobyl have demonstrated a correlation between low-dose radiation exposure and induced cataracts [6]. Klein et al. reported that subjects with diagnostic X-ray exposure have higher incidence of posterior subscapular cataracts [7]. There has been growing concern regarding lens opacities and cataract formation in relation to radiation exposure in the interventional radiology setting. 

There is also demonstrable evidence within the past decade for a genetic component of cataract development, which further elucidates some of the mechanisms behind DNA damage and response and repair pathways [8]. These genetically predisposed mechanisms have been discovered in animal models, and to go into depth at this time is beyond the scope of this narrative review.

Vano et al. has reported that lens injuries can occur to the interventionist and other medical staff if proper radiation safety precautions are not employed [9]. The ICRP and the United States National Council on Radiation Protection (NCRP) have formulated guidelines and standards regarding lens opacities and pathology based on deterministic radiation-induced effects [5, 10]. These radiation-induced effects appear only if a dose threshold is exceeded [5, 10]. As suggested by Ainsbury et al. [6], these standards have come under question in several journals, and the actual threshold may be less than previously estimated. With fluoroscopically guided procedures, the interventional pain physician is in close proximity to the patient. With this proximity, the interventionist faces increased exposure due to scatter radiation which can reach up to 6 feet from where the X-ray beam enters the patient [4].

## 2. Materials and Methods

Review of relevant data sources on radiation safety, occupational radiation exposure, and primary studies evaluating the effects of radiation exposure to the human eye, cataracts, and lens opacity were performed. Data sources included relevant literature in the English language, identified through searches on PubMed, and MEDLINE from 1966 to the present. The keywords radiation, cataracts, fluoroscopy, cataractogenesis, and lens opacity were entered into the Pubmed/Medline search engine to retrieve relevant journal articles. Manual searches of bibliographies of these review articles, primary articles, and abstracts were also performed.

### 2.1. ICRP Guidelines

It is important to review the ICRP's role in radiation safety, as well as to define the accepted nomenclature involved when describing the units of radiation.

The ICRP is an independent international organization which publishes quarterly articles that address fundamental recommendations regarding radiological protection [5]. In March of 2007, the ICRP updated their recommendations on safety and protection from ionizing radiation exposure [5]. The ICRP uses the International System of Units when publishing their recommendations. A Gray (Gy) is an SI unit of absorbed radiation dose of ionizing radiation [5]. A Sievert (Sv) is an SI unit of equivalent dose, effective dose, and operational dose quantities [5]. They are both equal to one joule per kg (=1 J kg^−1^). One Gy and Sv are equal to 100 Radiation Absorbed Dose (Rad) and 100 Radiation Equivalent in Man (REM), respectively [11, 12]. The nomenclature can be confusing, and is clarified in a review of radiation safety by Fishman et al. [12].

The ICRP-reported threshold for detectable opacities for (1) acute single exposure is 0.5–2.0 Sv (50–200 REM), (2) for highly fractionated or protracted exposures is 5Sv (500 REM), and (3) for annual dose rate for yearly highly fractionated or protracted exposure for many years is >0.1 Sv (10 REM) per year [13, 14] (Tables 1 and 2).

The ICRP threshold dose value for visual impairment (cataract) for (1) acute single exposure is 5 Sv (500 REM), (2) for highly fractionated or protracted exposures is >8 Sv (>800 REM), and (3) annual dose rate for yearly highly fractionated or protracted exposure for many years is >0.15 Sv/year (>15 REM/year) [5, 13] (Tables 1 and 2).

These ICRP thresholds are not without controversy either. There have been several epidemiological studies of low-dose radiation exposure and its association with cataracts in a variety of contexts. This is seen not only in the medical environment, but also in environmental and occupational exposure. The differentiation of cataracts and the relation to specific radio-particle exposure is beyond the realm of this article.

### 2.2. Cataracts and Lens Opacities

The various contexts include a spectrum spanning survivors from Hiroshima and Nagasaki to children living in contaminated territories of Chernobyl, as well as astronauts, pilots, and radiology technicians [6, 15–17]. However, with reported incidences of lens opacities and cataracts in these various contexts with alleged radiation doses less than these aforementioned thresholds, there is growing concern in the medical community that perhaps there is a nonthreshold effect for these cataracts [18]. Given this data, it is also important to note that ICRP guidelines and values have not significantly changed in the past 20 years [5].

Specifically, there is very little epidemiological data regarding the risk of lens opacification and cataract formation in the field of interventional radiology, much less spine or pain intervention. There have, however, been a few small studies looking at interventional radiologists and interventional cardiologists suggesting an increased prevalence of cataracts in this particular occupation [19–21].

Few studies have investigated average radiation exposure times and amount per procedure in an interventional pain setting. There has been an increasing number of case reports of injuries to the lens of the eye in operators and assistants performing interventional pain procedures [22]. In private practice, Manchikanti et al. reported radiation exposure time on average of 7.7 ± 0.21 seconds per interventional pain procedure [22]. The types of procedures performed included facet injections, transforaminal epidurals, caudal epidurals, and selective nerve blocks [22]. 

Botwin et al. reported average radiation exposure per procedure at the “glasses” badge for caudal epidural steroid injections, lumbar transforaminal epidural steroid injection, and lumbar discography as 2.47 mREM, 0.4 mREM, and 1.49 mREM, respectively, [11, 23, 24]. The “glasses” badge refers to a dosimeter placed directly outside and adjacent to the glasses to more accurately capture radiation exposure towards the eyes [11, 23, 24]. 

If one were to speculate in an interventional pain practice that the overall exposure, on average, is less than 2.0 mREM per procedure, and that an active practitioner were to perform an average of 40 procedures per week, the cumulative yearly radiation exposure would still be at least 10-times lower than the annual radiation exposure limits to the eye lens as suggested by the ICRP [5].

However, there are some shortcomings with this assumption. This “average” only takes into consideration a very small array of procedures in the repertoire of most interventional pain physicians. This speculation does not take into consideration more advanced procedures that may require “live” beam time such as spinal cord stimulation implants/trials, intrathecal pump implants, and vertebroplasty/kyphoplasty. Other less invasive percutaneous procedures, such as sympathetic blockade, medial branch blocks, radiofrequency ablation, SI joint injections, MILD procedure, and percutaneous fusions have not been considered.

There are also multiple variables that can influence the amount of lens exposure. For example, the distance the practitioner is from the patient, height of the practitioner, patient size, and weight, age, as well as the use of collimation techniques can alter the amount of scatter radiation emanating from the patient [12]. Vano et al. also reported that fluoroscopy hardware from different companies can produce varying dose rates [14].

Manchikanti et al. also further demonstrated that an interventional pain physician's experience plays a major factor in determining radiation exposure time and that with less experienced interventional pain physicians longer radiation exposure times were noted per interventional procedure [22]. In the academic setting, Zhou et al. also reported average radiation exposure time could range from 2- to 14-times higher than in a private practice setting depending on the type of interventional pain procedure [25]. The academic setting included attending physicians supervising the performance of in-training residents and fellows in a spine interventional procedure setting. In these settings, the complete amount of radiation exposure is not clear. It is also unclear what percentage of interventional pain physicians actually uses eye protection to help further decrease their risk. Various institutions, whether private practice or academia, do not seem to clearly have defined standard protocols in radiation safety in the United States.

### 2.3. Eye Protection

There is little question as to whether appropriate eyewear decreases radiation transmission to the actual eye. Cousin et al. and Richmond et al. have both demonstrated decreased transmission rates of up to 70% and 98%, respectively [26, 27]. As previously mentioned, primary scatter emanating from the patient is a source of unwanted exposure to the eye. In addition, radiation exposure can also emanate from the interventionist's head and “rescatter” to the eye. Cousin et al. acknowledge these possibilities and emphasizes the importance of proper positioning of the interventionist's head [26]. For example, the interventionist's head may be oriented 90 degrees to the scatter orientation when viewing the monitor (parallel to the prone/supine patient) [26]. If the interventionist's eyewear does not have protected side shields, the eyes could receive a significant portion of scatter radiation [26]. It is extremely vital for the interventionist to be cognizant of his/her surroundings. This reiterates the importance of increasing the distance between the physician and the source of the radiation, it also emphasizes decreasing the amount of exposure time, which can both drastically reduce unnecessary radiation via scatter. 

How much cataract risk reduction occurs with the use of protective eyewear is not clear. The O'CLOC study is currently evaluating both French interventional cardiologists and noninterventional cardiologists to further delineate occupational exposure, classify these exposures, and to further shed light on cataract formation risk in the interventional group [15]. The questionnaire used in the study will also survey the use of lead eye glasses amongst the interventional cardiologists. This information may help determine actual risk reduction for cataracts in those wearing protective eyewear. These results are expected to be released in 2011 [15].

## 3. Discussion

According to the ICRP, the risk of cataract formation in the context of occupational radiation exposure is based on deterministic radiation-induced effects [5]. Although the ICRP's threshold dose values for both lens opacities and cataract formation are clearly defined in Tables 1 and 2, there have been numerous epidemiological studies and articles that suggest there is a non-threshold effect that falls well below these dose values. In the context of the practicing pain interventionist, Manchikanti et al. and Botwin et al. have reported average reported radiation times and average radiation exposure per procedure, respectively. Under these practicing circumstances, the predicted radiation exposure in a busy interventional pain practice would still be well below the threshold dose values set forth by the ICRP for lens opacities or cataract formation. However, these practicing circumstances are not realistic, as these scopes of procedures do not represent the true spectrum performed across practices in the community. Furthermore, depending on the type of setting (private practice versus academia), as well as the experience of the practitioner, the average radiation times and average radiation exposure can be highly variable. This high variability and the inherent uniqueness of the thousands of practices in the United States make it very difficult to accurately assess and define the risks involved to the interventionist and the possible damage to the eye and lens.

Furthermore, one cannot simply ignore the implication of the multiple epidemiological studies and the documented cases of cataract formation in “controlled” interventional laboratories. There is no question about the effectiveness of appropriate leaded (or other high atom containing lenses) lenses in reducing the transmission percentage of radiation exposure. There is, however, no clear evidence examining how effective these measures are in reducing risk of cataract or lens opacity formation.

The scope of this review is not only to identify the apparent occupational radiation risk and the lack of information regarding it, but also to recognize some potential studies on the horizon that may provide invaluable insight. The O'CLOC study evaluating French interventional cardiologists versus noninterventional cardiologists may provide such insight [15].

## 4. Conclusion

In conclusion, based on this extensive review of the literature involving risk of eye/lens pathology and its relation to occupational radiation exposure to the interventionist, further research is needed to help further define these risks. Nevertheless, it is the authors' recommendation for the interventional pain practitioner to use shielded eyewear until further research can clearly elucidate the short- and long-term risks of occupational radiation exposure.

## Figures and Tables

**Table 1:** ICRP threshold dose values for damage to the eyes in Sv and REM [5, 13].

		Acute single exposure	Highly fractionated or protracted exposures	Annual dose rate for early highly fractionated or protracted exposures for many years
Detectable opacities	SI unit	0.5–2.0 Sv	5 Sv	>0.1 Sv per year
	Conventional unit	50–200 REM	500 REM	>10 REM/year
Visual impairment (cataract)	SI unit	5 Sv	>8 Sv	>0.15 Sv (150 mSv/year)
	Conventional unit	500 REM	800 REM	>15 REM/year

**Table 2:** ICRP threshold dose values compared to observed opacities and cataracts [5, 13].

		ICRP	Observed cases
Detectable opacities	SI unit	0.5–2.0 Sv	<1 Sv
	Conventional unit	50–200 REM	<100 REM
Visual impairment (cataract)	SI unit	5 Sv	<1 Sv
	Conventional unit	500 REM	<100 REM
